# Safety and Efficacy of Peptide Receptor Radionuclide Therapy (PRRT) Following Bland Embolization for Metastatic Neuroendocrine Tumors

**DOI:** 10.3390/cancers16152703

**Published:** 2024-07-30

**Authors:** Adam Alayli, Hoang Ngo, Dhiraj Sikaria, Altan Ahmed, Elias Salloum, Jonathan R. Strosberg, Taymeyah E. Al-Toubah, Bela Kis, Mintallah Haider, Ghassan El-Haddad

**Affiliations:** 1 University of South Florida Morsani College of Medicine, Tampa, FL 33602, USA; adamalayli@usf.edu (A.A.); hngo51@usf.edu (H.N.); dsikaria@bidmc.harvard.edu (D.S.); 2 Department of Diagnostic Imaging and Interventional Radiology, Moffitt Cancer Center, Tampa, FL 33612, USA; altan.ahmed@moffitt.org (A.A.); elias.salloum@moffitt.org (E.S.); bela.kis@moffitt.org (B.K.); ghassan.elhaddad@moffitt.org (G.E.-H.); 3 Department of Gastrointestinal Oncology, Moffitt Cancer Center, Tampa, FL 33612, USA; taymeyah.al-toubah@moffitt.org (T.E.A.-T.); mintallah.haider@moffitt.org (M.H.)

**Keywords:** bland embolization, peptide receptor radionuclide therapy, hepatotoxicity, neuroendocrine tumor

## Abstract

**Simple Summary:**

Peptide receptor radionuclide therapy using ^177^Lu-DOTATATE is a standard therapy for patients with metastatic neuroendocrine tumors. Many patients with liver-dominant disease undergo liver embolization as an earlier line of treatment. It is unclear whether prior embolization impacts the safety and efficacy of subsequent ^177^Lu-DOTATATE treatment. We analyzed 171 patients who underwent treatment with ^177^Lu-DOTATATE, among whom 61 underwent prior embolization. There was no significant difference in serious liver toxicity or radiographic progression of liver tumors between the group that had undergone prior embolization and the group that had not. The conclusion is that peptide receptor radionuclide therapy with ^177^Lu-DOTATATE in patients who had prior liver embolization is both safe and effective.

**Abstract:**

**Rationale**: Evaluating the long-term safety and efficacy of peptide receptor radionuclide therapy (PRRT) in patients with metastatic neuroendocrine tumors (mNETs) who have undergone prior bland hepatic transarterial embolization (TAE). **Methods**: Retrospective review of mNET patients who received PRRT with ^177^Lu-DOTATATE between 4/2018 and 02/2022 with and without prior TAE. The most recent clinical, imaging, and laboratory findings, including hepatic Common Terminology Criteria for Adverse Events v5.0, were compared to pre-PRRT. **Results**: 171 patients (95 M, 76 F, median age = 66) with mNET of different primary sites (9 foregut, 100 midgut, 9 hindgut, 44 pancreas, 9 unknown) received at least 1 cycle of PRRT with at least 6 months of follow-up, 110 of whom were embolization-naïve and 61 who had prior TAE. The median follow up was 22 months (range: 6–43). Patients with prior TAE had higher liver tumor burden on average than patients without prior TAE; however, the difference was not statistically significant (*p* = 0.06). There was no significant difference in the rates of G3 or G4 hepatotoxicity (*p* = 0.548 and *p* = 0.999, respectively) in patients who underwent prior TAE and those who were TAE-naïve. The hepatic progression-free survival was 22.9 months in TAE-naïve patients and 25.7, 20.2, and 12.8 months in patients with 1, 2, and 3 prior TAE treatments, respectively. **Conclusion**: Peptide receptor radionuclide therapy following transarterial bland embolization for mNET is safe and effective.

## 1. Introduction

The liver is the most common site of distant metastases among patients with well-differentiated neuroendocrine tumors (NETs) [1,2]. Surgical cytoreduction may be an option for patients with limited hepatic disease burden. Most patients with unresectable somatostatin receptor (SSTR)-positive tumors are initially treated with somatostatin analogs (SSAs). Systemic and intra-arterial therapies are options for patients who have progressive liver-dominant metastases [3,4].

Peptide receptor radionuclide therapy (PRRT) is a standard second-line systemic treatment for progressive SSTR-positive tumors. The phase III NETTER-1 trial demonstrated a clinically and statistically significant improvement in progression-free survival (PFS) with the radiolabeled somatostatin analog ^177^Lu-DOTATATE, compared to high-dose octreotide in patients with progressive midgut NETs [5]. The objective response rate was 18%. In patients with pancreatic NETs, higher objective response rates have been reported, up to 40%, although phase III data are still lacking [6].

Hepatic transarterial embolization (TAE) is a treatment generally offered to patients with well-differentiated liver-dominant metastases who are not surgical candidates, to either debulk their tumor or for those with symptomatic or progressive disease despite the use of a somatostatin analog [7].

Systemic treatment options for metastatic pancreatic NETs include everolimus, sunitinib, cytotoxic chemotherapy (e.g., capecitabine and temozolomide), ^177^Lu-DOTATATE, as well as hepatic TAE in patients with liver-dominant disease [8,9,10]. Options for midgut NETs with carcinoid syndrome are generally more limited: either hepatic transarterial embolization or ^177^Lu-DOTATATE.

Understanding the toxicity profile of ^177^Lu-DOTATATE after hepatic transarterial embolization is paramount to ascertain whether PRRT can be safely administered after liver embolization. There is a paucity of data regarding the effects of TAE prior to PRRT. We sought to analyze the risk of hepatic and non-hepatic toxicities in patients who received treatment with ^177^Lu-DOTATATE, comparing those who had undergone prior TAE to patients who were embolization-naïve. We also compared the efficacy of ^177^Lu-DOTATATE in both groups of patients.

## 2. Materials and Methods

**Figure 1:**
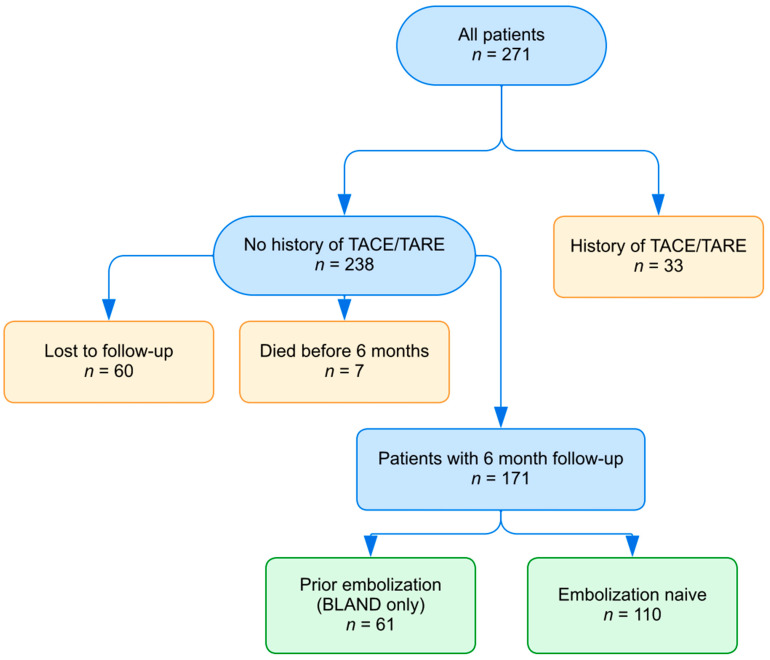
Flowchart showing patient selection.

We conducted a retrospective single-institution study that reviewed all patients who received ^177^Lu-DOTATATE at Moffitt Cancer Center from April 2018 to February 2022 due to a metastatic neuroendocrine tumor. Institutional review board approval was acquired from the Institutional Review Board of the University of South Florida (Tampa, FL, USA), and waiver of consent was granted due to the study’s retrospective nature. Patients were excluded if they had history of transarterial chemoembolization (TACE) or radioembolization (TARE) prior to PRRT, irrespective of their history of TAE (Figure 1). This was performed to preserve the consistency of our data and eliminate any possible room for bias or confounders in our dataset. Additionally, there is an absence of reported data in the literature regarding the superiority of one type of embolization over the other, so it is our institutional preference to perform TAE. There was no restriction on the number of embolization procedures or number of PRRT cycles.

Laboratory parameters, including creatinine, bilirubin, albumin, ALT, AST, ALP, and platelet count, were evaluated at the following timepoints: immediately before the first round of PRRT and at the latest follow-up, if available, at least six months after the final round of PRRT. The Common Terminology Criteria for Adverse Events v5.0 was used to grade toxicities. Clinical findings, imaging, and adverse events using the Common Terminology Criteria for Adverse Events v5.0 were analyzed. Finally, global progression-free survival (PFS) and hepatic progression-free survival (hPFS) after the final PRRT cycle were calculated based on chart and imaging review (radiographic documentation of progression leading to change in therapy). Statistical analysis was conducted using Fisher’s exact testing for cohort comparisons and log-rank tests for survival analysis.

## 3. Results

Overall, 238 patients (126 male, 112 female; median age = 66 years) received at least 1 cycle of PRRT for metastatic well-differentiated NETs between April 2018 and February 2022. Primary sites included the gastroduodenum, midgut, colorectum, pancreas, lung, and unknown primary. Table 1 describes the patient demographics. Most patients (*n* = 155) had not undergone prior TAE, while 83 patients received prior TAE. In total, 7 patients died (prior embolization: 2; embolization-naïve: 5) and 60 were lost to follow-up (prior embolization: 20; embolization-naïve: 40) prior to the 6-month follow-up timepoint after their last dose of PRRT. This left 171 patients with a follow-up of at least 6 months after their last dose, including 61 of 83 (73%) with prior-TAE and 110 of 155 (71%) of the TAE-naïve patients (Figure 1). There was no significant difference in the proportion of prior-TAE and TAE-naïve patients in the excluded and included cohorts (*p* = 0.68). The median time from embolization to first PRRT was 2 years (range: 0.3–14 years, IQR: 1.05–2.95) and the median follow-up time after the last dose of PRRT for patients with at least 6-month follow-up was 21 months (range: 6–43, IQR: 11–32). The average number of PRRT cycles was 3.7 for the prior-TAE cohort and 3.8 for the TAE-naïve cohort. Although some patients received different numbers of PRRT cycles for various reasons, there was no statistically significant difference in the average number of cycles received in the prior-TAE compared to the TAE-naïve group (*p* = 0.3).

Hepatic tumor burden was assessed via pre-PRRT cross-sectional imaging and DOTATATE-PET scan. In the cohort who underwent prior TAE, liver tumor burden was <25% in 40 patients (66%), 25–50% in 16 patients (26%), and >50% in 5 patients (8%). Among the embolization-naïve group, hepatic tumor burden was <25% in 90 patients (82%), 25–50% in 15 patients (14%), and >50% in 5 patients (5%). On average, the cohort with prior TAE had a higher hepatic tumor burden than those without prior embolization, although this was not statistically significant (*p* = 0.06). Six patients (10%) in the previous TAE cohort developed grade 3 hepatotoxicity (AST: 1 patient, ALT: 0, ALP: 2, bilirubin: 3). One patient in this group developed transient grade 4 hyperbilirubinemia, with resolution in 2 weeks. Similar rates of hepatotoxicity occurred in the embolization-naïve group. Seven patients (6%) developed grade 3 hepatotoxicity (AST: 3 patients, ALT: 1, ALP: 5, bilirubin: 2) and 2 patients experienced grade 4 hepatotoxicity (one patient with hyperbilirubinemia and one with elevated AST). The patient with grade 4 hyperbilirubinemia had a pre-treatment history of grade 3 and 4 hepatotoxicity. There was no statistically significant difference in the incidence of grade 3 (*p* = 0.548) or grade 4 (*p* = 0.999) hepatotoxicity between the two cohorts (Table 2).

**Figure 2:**
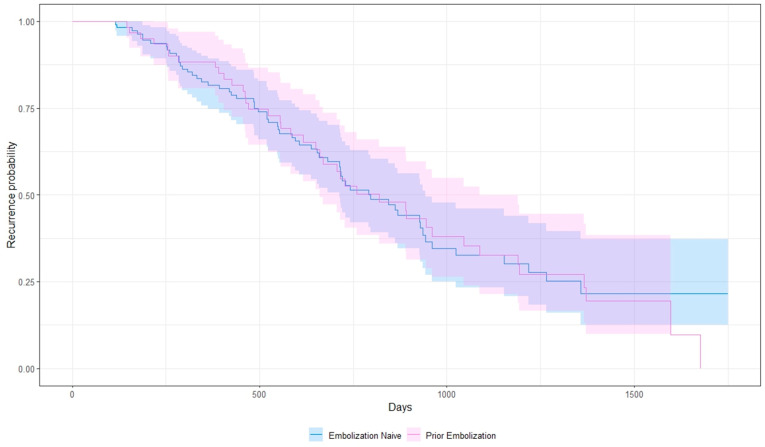
Kaplan–Meier curve showing hepatic progression-free survival.

**Figure 3:**
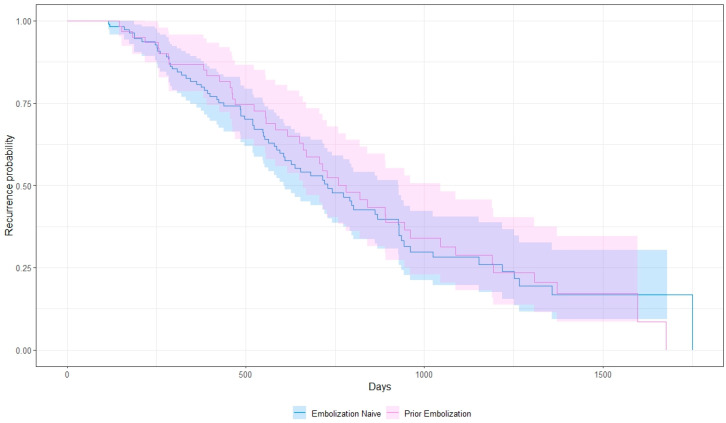
Kaplan–Meier curve showing global progression-free survival.

Among embolization-naïve patients, the median hepatic progression-free-survival (hPFS) was 22.9 months (range: 3.8–58.3), and the median global progression-free-survival (PFS) was 22.4 months (range: 3.8–58.3). Patients with at least 1 prior cycle of embolization, defined as one or more treatments needed to treat the entire tumor burden, had a median hPFS and PFS of 24.7 (range: 4.8–56, *p* = 0.89) and 24.2 months (range: 4.8–56, *p* = 0.81), respectively (Figure 2 and Figure 3). When analyzed based on number of cycles, patients with only 1 prior TAE cycle had a median hPFS of 25.7 months (range: 4.8–56, *n* = 52), those with 2 had a median hPFS of 20.2 months (range: 13–27.3, *n* = 7), and those with 3 had a median hPFS of 12.8 months (range: 7.1–18.5, *n* = 2). One patient was excluded due to an unknown number of prior embolizations. Additionally, 25 patients discontinued PRRT mid-course due to progression of disease or cytopenias. Five had G3 cytopenias. Twelve (20%) of the patients who discontinued treatment early had prior embolization and the remaining thirteen did not (12%), with no significant difference between both cohorts (*p* = 0.16).

## 4. Discussion

Several studies have established a low risk of hepatotoxicity after PRRT. Kwekkeboom et al. analyzed over 500 patients who received ^177^Lu-DOTATATE and found transient hepatotoxicity in 2 patients, with no long-term toxicity [11]. Similarly, Jiang et al. followed 30 patients treated with ^177^Lu-DOTA-EB-TATE, of whom only 1 developed G3 hepatotoxicity [12].

The safety and efficacy of ^177^Lu-DOTATATE in patients who have had prior TAE is unclear. Hamiditabar et al. evaluated the risk of hepatotoxicity after PRRT in patients who had received prior bland embolization, TACE, or TARE and found no statistically significant difference in toxicity when comparing embolization-naïve patients to patients who had previously undergone embolization [13]. Our large, single-institution retrospective study with long-term follow-up suggests that the risk of hepatotoxicity remains low, and that efficacy remains high in patients treated with ^177^Lu-DOTATATE who have undergone prior TAE. We did not observe any significant difference in median hPFS in patients who had prior TAE compared with patients who were embolization-naïve. Patients who had multiple prior embolizations experienced a shorter hPFS after PRRT, although the small sample size makes it difficult to draw any conclusions. More data would be needed to establish whether this indicates a more aggressive disease biology.

Other studies have examined combination therapies in a differing sequence than reviewed here. Braat et al. followed patients who received radioembolization with yttrium-90 or holmium-166 microspheres after completing their PRRT in order to investigate the hepatotoxicity and safety of these combination therapies [14,15]. In the Y-90 study, biochemical and hematological toxicity were observed in 26% of patients after 3 months, although the number of total patients with G3 or G4 hepatotoxicity remained relatively stable when compared to baseline. Additionally, 11% of patients had complications related to radioembolization, such as gastric ulceration, radiation pneumonitis, cholangitis, or liver abscesses, and 1 patient died due to radioembolization-induced liver disease. The post-PRRT Ho-166 radioembolization study reported similar results, with 1 fatal case of radioembolization-induced liver disease. It is unclear whether previous PRRT had a significant contribution to the fatal hepatic toxicity.

This study has several limitations. First, its retrospective nature makes it susceptible to selection bias. Additionally, this study only addresses the combination of bland embolization with PRRT, since most qualifying patients at our institution received bland embolization as opposed to other types of embolization. Also, this study does not fully address the impact of PRRT with prior embolization on a patient’s overall survival (OS) because we did not control for other systemic therapies the patients may have received. Lastly, while our data suggest that PRRT can be safely sequenced after TAE, they do not imply that this sequence of therapies is necessarily optimal. Only a sequencing trial comparing PRRT after bland embolization, or vice versa, with an endpoint of progression-free survival can answer this question. Such a study would require a very large sample size with a long duration of follow-up.

## 5. Conclusions

PRRT can be safely administered to patients who have previously undergone hepatic transarterial bland embolization. These patients are not at increased risk of developing high-grade or long-term hepatotoxicity. Moreover, there is no evidence of a worsened hepatic or global PFS after ^177^Lu-DOTATATE in patients with prior bland TAE.

## Figures and Tables

**Table 1:** Demographic information and patient characteristics.

	Prior Embolization (*n* = 61)	Embolization-Naïve (*n* = 110)
**Gender**		
Male	34 (56%)	61 (56%)
Female	27 (44%)	49 (44%)
**Age at PRRT**		
Mean (SD)	64.2 (10)	64.4 (8.9)
**Follow-up Time (months)**		
Mean (SD)	22.4 (11.4)	20.9 (10.4)
**Years From Diagnosis to PRRT**		
Mean (SD)	7.2 (5.5)	6.4 (5.8)
**Primary Tumor Location**		
Foregut	3 (5%)	6 (5%)
Midgut	43 (71%)	57 (52%)
Hindgut	2 (3%)	7 (6%)
Pancreas	11 (18%)	33 (30%)
Other/Unknown	2 (3%)	7 (7%)
**Tumor Grade**		
1	19 (31%)	42 (38%)
2	29 (48%)	52 (47%)
3	8 (13%)	6 (6%)
Uncertain	5 (8%)	10 (9%)
**Tumor Burden**		
<25%	40 (66%)	90 (82%)
25–50%	16 (26%)	15 (14%)
>50%	5 (8%)	5 (5%)
**PRRT Cycles**		
1	0 (0%)	3 (3%)
2	5 (8%)	4 (4%)
3	6 (10%)	10 (9%)
4	50 (82%)	93 (84%)

Abbreviations: PRRT = peptide receptor radionuclide therapy.

**Table 2:** Overall toxicity comparisons between patients with and without pre-PRRT bland embolization show no significant difference in long-term toxicity.

Toxicity Metric	Prior Embolization	Embolization-Naïve	*p*-Value
Patients with grade 3 hepatic adverse events	6	7	0.548
Patients with grade 4 hepatic adverse events	1	2	0.999

## Data Availability

The data presented in this study is available on request from the corresponding author.
